# Supplementary material for: Learning curve and surgical outcome of robotic assisted colorectal surgery with ERAS program
Source: Sci Rep. 2022 Nov 29;12:20566. doi: 10.1038/s41598-022-24665-w (PMC9709162; doi:10.1038/s41598-022-24665-w)
Supplement: Supplementary file 1 — Supplementary Information 1. [file 41598_2022_24665_MOESM1_ESM.zip › LSC Raw data-Ñ[▒K/125/N_ñw╝╨░O▒Kñσ.pdf]

W52 / W52

臺中榮民總醫院
手術室護理記錄

[redacted]

基本資料

- 室別： 二醫手術室-31室，手術科別： CRS，手術類別： 常規，日期： 2021/04/14
- 進入時間： 1210 ； 麻醉開始時間： 1220 ； Time Out時間： 1320 ； 劃刀時間： 1321 ； 手術結束時間： 1625 送出時間： 1630 ；
- 麻醉方式： GE

  診斷：S colon tumor 20 cm FAV, sonic cission.

  手術名稱：Laparoscopic Anterior resection and anastomosis - malignant

  手術局部用藥：nil

  術前預防性抗生素：
  - 抗生素名稱一： Veterin 1GM IVD由麻醉護理師給予
  - 時間： 1250
  - 上次簽章: 梁凱雯

  ，追加預防性抗生素：
- 手術醫師： 林俊余，楊曙榮，陳勇先
- 麻醉醫師： VS 賴慧卿
-
  - 刷手護士A ： 李庭，時間： 1210，接替刷手一： 羅渝軒，時間： 1300
  - 流動護士A ： 黃鈺琇，時間： 1210，接替流動一： 梁凱雯，時間： 1300

皮膚狀況

A-V Shunt： 無

術前： 完整

記錄者： 上次簽章: 黃鈺琇

術後： 不完整

- 7. 紅痕

部位(術後)：

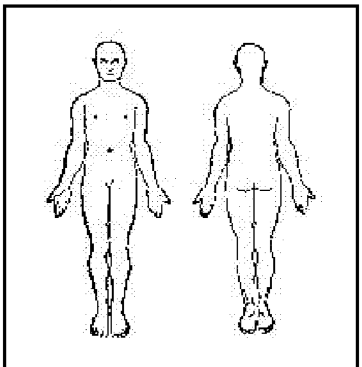

前胸5cmX30cm紅壓可白，右上臂內側1cmX10cm紅痕

記錄者： 梁凱雯

手術準備

手術臥式 單一手術臥式

-
  - 手術臥式： 截石臥式
  - 使用附件： 溫毯 38 ℃，頭圈，腳架，其它： 頭架
  - 皮膚準備： 10%Aq Betadine，Hibiscrub，2% Chlorhexidine
  - 電燒： 使用電燒: 電極片位置： 其它： 床上迴路電極板，雙極電燒，記錄者： 上次簽章: 黃鈺琇

止血帶T： 無

植入物

無

標本送檢

- 細菌培養 無
- 細胞學 無
- 冰凍切片 無
- 病理標本
  組織名稱一： S-colon
  ,
  組織名稱二： distal cut end
  ,
  組織名稱三： IMA root
- 截肢 無

標本交付 標本： 參 袋 , 標本： 零 盒 , 標本： 零 瓶 , 細胞學： 零 管 , Culture Tube： 零 管 , 被交付醫師： R 陳勇先 ,

- 記錄者： 梁凱雯
- 時間： 1630

傷口術後敷料

傷口敷料： 傷口敷料： 無

- 請於圖上標示部位：

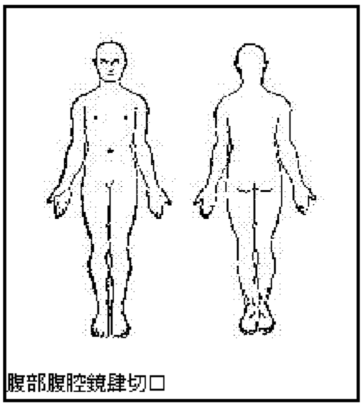

- 傷口敷料： mepore

導管

導管	天數	導管資訊	插入日期	插入時間	更換日期	拔除日期	拔除時間	拔除原因	預計拔除/更換	預計拔除/更換日期
Sump NG Tube(1)	1	號碼:16 Fr.; 部位:胃; 註記:fix65cm;	2021/04/14	1310						
Foley(1)	1	號碼:16 Fr.; 部位:膀胱;	2021/04/14	1315						

用物計數

- 手術中出血量： minimal c.c.
- 手術中尿量： 200 c.c.
-
  - 其它： 無

用物計數： 多切口同時縫合

- 1.器械： 正確 , 2.縫針： 正確 , 3.刀片： 正確 , 4.紗布： 正確 , 5.腹部墊： 未使用 , 6.紗球： 未使用 , 7.PEANUTS ： 未使用 , 8.紗條： 未使用 , 9.絲帶： 未使用 , 10.針頭： 未使用 , 11.Vessel loop： 未使用 , 12.Boots： 未使用 , 13.Jelco： 未使用 , 14.棉條： 未使用 ,
  - 第一次：
    - 刷手護士 ： 羅渝軒
    - 流動護士： 上次簽章: 梁凱雯
    - 時間： 1515

  ,
  - 第二次：
    - 刷手護士 ： 羅渝軒
    - 流動護士： 上次簽章: 梁凱雯

- 時間： 1525

,

- 第三次：
  - 刷手護士 ： 羅渝軒
  - 流動護士： 上次簽章: 梁凱雯
  - 時間： 1535

備註

備註：NIL

- 記錄者： 梁凱雯
- 時間： 1630

填寫護理人員 梁凱雯

正式摘要

W52 / W52

doctorRank:B
